# Genotyping-in-Thousands by sequencing panel development and application to inform kokanee salmon (*Oncorhynchus nerka*) fisheries management at multiple scales

**DOI:** 10.1371/journal.pone.0261966

**Published:** 2021-12-23

**Authors:** Sarah L. Chang, Hillary G. M. Ward, Michael A. Russello

**Affiliations:** 1 Department of Biology, University of British Columbia, Kelowna, BC, Canada; 2 British Columbia Ministry of Forests, Lands, Natural Resource Operations and Rural Development, Penticton, BC, Canada; University of Iceland, ICELAND

## Abstract

The ability to differentiate life history variants is vital for estimating fisheries management parameters, yet traditional survey methods can be inaccurate in mixed-stock fisheries. Such is the case for kokanee, the freshwater resident form of sockeye salmon (*Oncorhynchus nerka*), which exhibits various reproductive ecotypes (stream-, shore-, deep-spawning) that co-occur with each other and/or anadromous *O*. *nerka* in some systems across their pan-Pacific distribution. Here, we developed a multi-purpose Genotyping-in-Thousands by sequencing (GT-seq) panel of 288 targeted single nucleotide polymorphisms (SNPs) to enable accurate kokanee stock identification by geographic basin, migratory form, and reproductive ecotype across British Columbia, Canada. The GT-seq panel exhibited high self-assignment accuracy (93.3%) and perfect assignment of individuals not included in the baseline to their geographic basin, migratory form, and reproductive ecotype of origin. The GT-seq panel was subsequently applied to Wood Lake, a valuable mixed-stock fishery, revealing high concordance (>98%) with previous assignments to ecotype using microsatellites and TaqMan^®^ SNP genotyping assays, while improving resolution, extending a long-term time-series, and demonstrating the scalability of this approach for this system and others.

## Introduction

Freshwater fish populations have declined 83% over the past forty years as a result of the cumulative effects of overfishing, habitat degradation, climate change, dams and other migration barriers [1–3]. Today, fisheries managers face challenges maintaining a balance between harvesting commercially or recreationally valuable stocks and conserving productive populations in fisheries with diverse demographics [4, 5]. Mixed-stock fisheries present a further challenge for management as stock specific parameters are required to set harvest targets while meeting spawning escapement goals [6].

For mixed-stock fish populations, traditional survey methods can be inaccurate due to overlaps in morphology between closely-related life history forms [7]. In response, genetic stock identification (GSI) techniques have been developed to assess stock proportions by delineating individuals by genetic origin and considering barriers to gene flow [8–10]. Genetic stock identification panels have previously featured allozymes [11], microsatellites [12], and mitochondrial DNA [13, 14] as genetic markers of choice, but suffer from limited statistical power for precise estimates of stock composition within some species [15, 16]. Recent advances in massively parallel DNA sequencing (also known as next generation sequencing; NGS) have made great strides in improving speed and reducing costs of obtaining genetic data, providing opportunities for the development and application of novel genotyping approaches ideal for fisheries management applications [17].

Genotyping-in-Thousands by sequencing (GT-seq) is a multiplex amplicon sequencing approach that is able to simultaneously generate genotypes for thousands of individuals at hundreds of single nucleotide polymorphism (SNP) markers [18]. This technique can increase the number and vary the composition of diagnostic loci in GSI panels relative to traditional markers, subsequently increasing the statistical power needed to distinguish sub-populations within the same system [19, 20]. Delineating mixed-stock fish populations requires careful consideration of candidate loci, taking into account detectable genetic variation and the level of tolerable error as key considerations [9, 21]. For example, loci exhibiting putative signatures of selection can provide more information than neutral loci for individual assignment, especially in systems with more recent divergence or where low levels of gene flow and consistent selection persist between co-occurring stocks [22].

Kokanee, the freshwater resident form of sockeye salmon (*Oncorhynchus nerka*), is a species of economic, ecological, and cultural value. Kokanee are the 3^rd^ most valuable freshwater fishery in British Columbia, Canada, represent a primary prey species for sustaining lucrative rainbow trout fisheries ($555 million/year in British Columbia), and serve important cultural and spiritual roles within some First Nations communities [23–26]. Kokanee have evolved multiple times independently across their pan-Pacific distribution [27], where three reproductive ecotypes have been observed including: 1) stream-spawners that move to tributaries to spawn in free-flowing water [28]; 2) shore-spawners that spawn in angular gravel along lake shores [29]; and 3) deep-spawners (also referred to as black kokanee) that spawn at depth in lakes [30]. Population genetic and genomic analyses have reconstructed neutral population structure largely by lake and geographic basin [27, 31], as well as revealing an underlying genetic basis associated with reproductive ecotypes and migratory forms [22, 32–35]. This underlying genetic divergence across geographic basin, migratory and reproductive ecotypes provides an excellent framework for developing a multi-scaled GSI tool for informing kokanee fisheries management, and can be used as a guide for the development of genetic panels for other fish species with co-occurring ecotypes such as lake trout (*Salvelinus namaycush*) [36].

Here, we developed a GT-seq panel for use in informing kokanee fisheries management at multiple scales (geographic basin, migratory ecotype, and reproductive ecotype). We validated the panel using sensitivity analyses and self-assignment across systems spanning three distinct geographical basins in British Columbia and comprising locations where migratory forms and reproductive ecotypes co-occur or reside in isolation. We subsequently genotyped kokanee individuals that were distributed across British Columbia to evaluate individual assignment accuracy and applied this new tool to assess stock proportions of co-occurring shore- and stream-spawning kokanee in Wood Lake, Canada’s highest-use wild stock kokanee fishery. More broadly, our results highlight important considerations when developing and applying GT-seq panels for fisheries management.

## Materials and methods

### British Columbia-wide GT-seq SNP panel design

**Fig 1:**
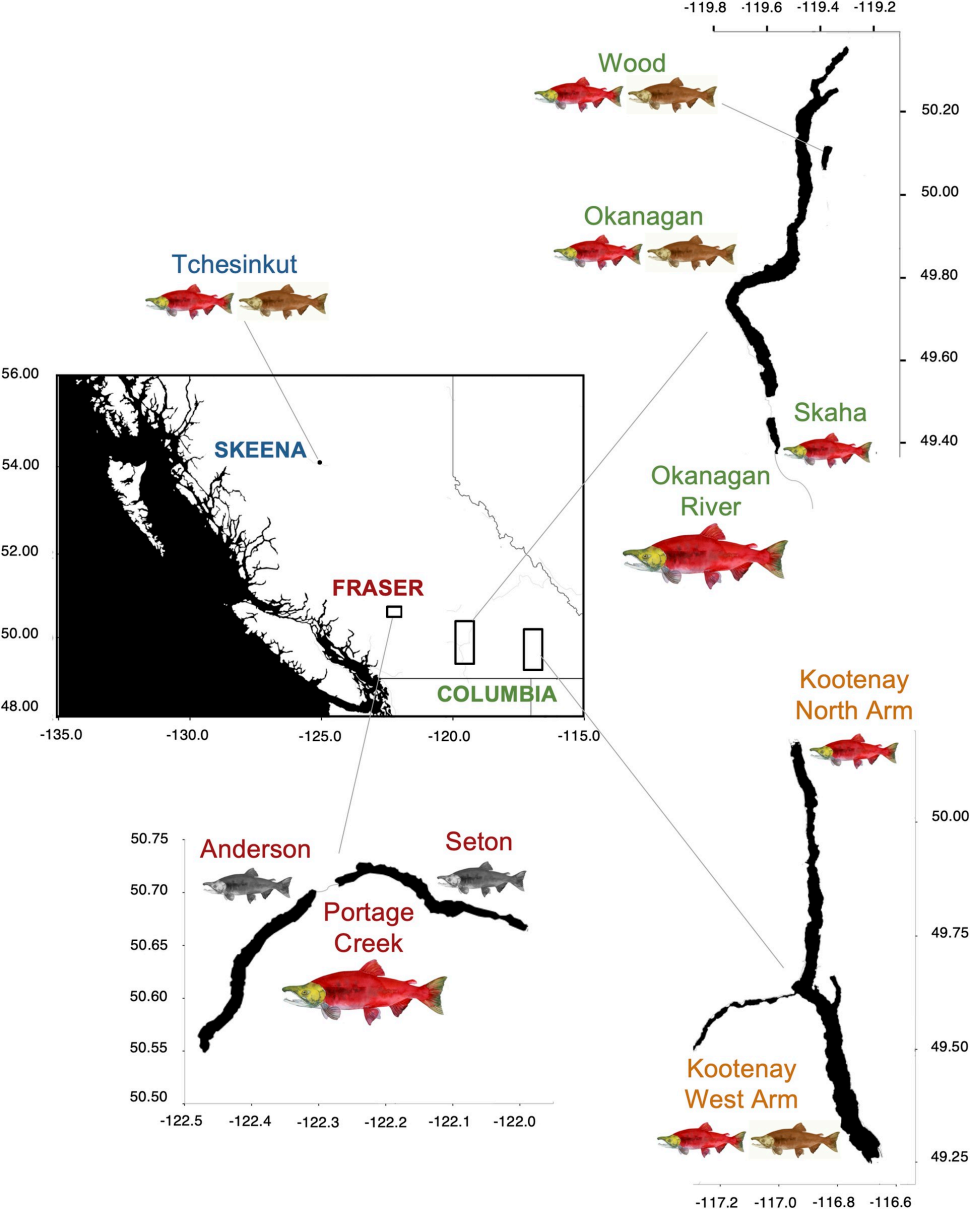
Map of British Columbia with baseline *Oncorhynchus nerka* populations used in initial panel design. Fish image size represents migratory form (large = anadromous sockeye salmon, small = resident kokanee) and color represents reproductive ecotype (red = stream-spawning, brown = shore-spawning, black = deep-spawning kokanee). Text color of population origin indicates historical drainage (green = Columbia River, red = Fraser River, blue = Skeena River, orange = Kootenay Lake kokanee that may be historically linked to either Columbia or Fraser drainages). All map units in decimal degrees. This figure was modified from [35].

**Fig 2:**
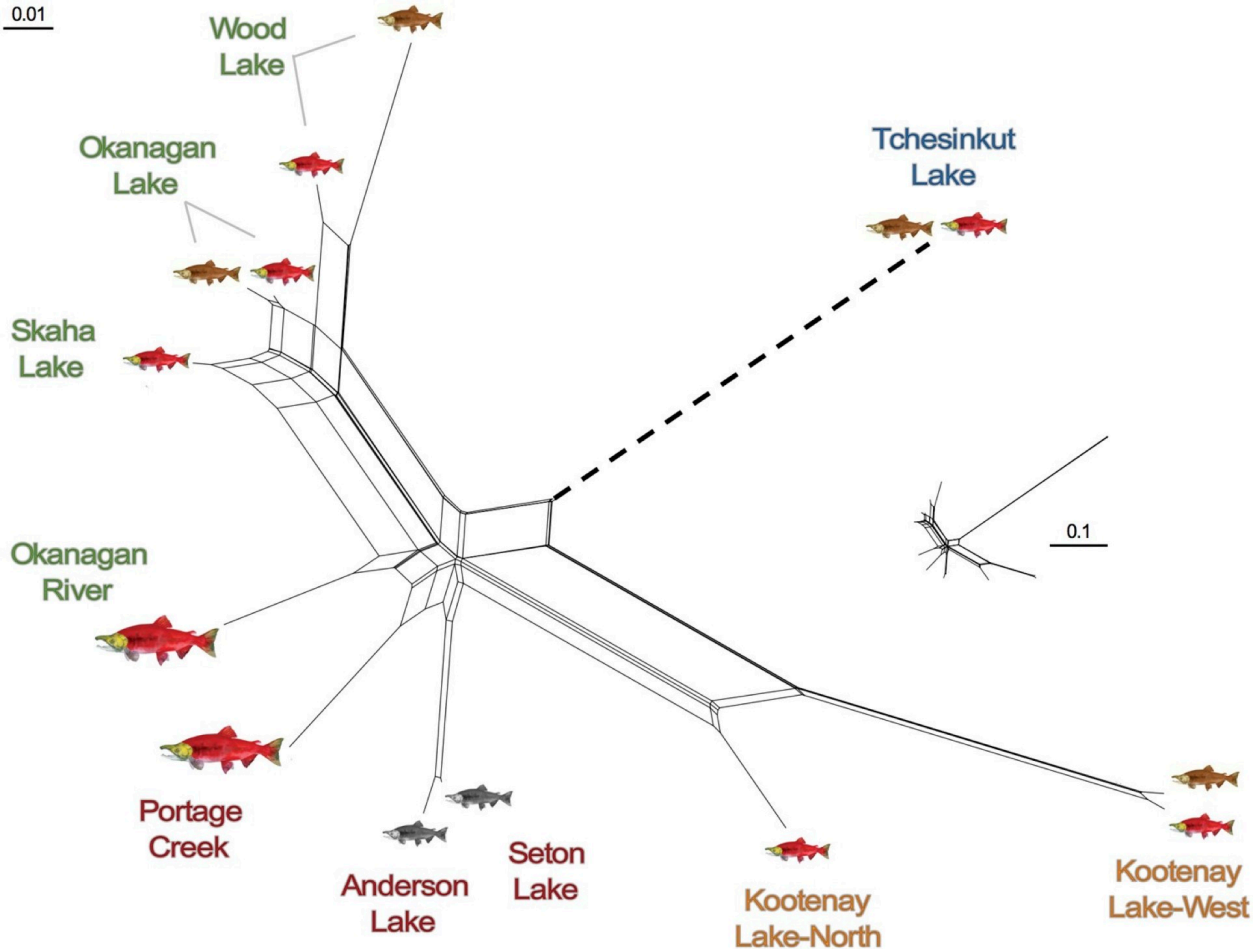
Two-dimensional NeighbourNet (Bryant and Moulton 2004) population network based on Weir and Cockerham’s (1984) unbiased estimates of pairwise genetic differentiation (*θ*) calculated using genotypic data at 6,234 neutral SNPs. Length of the Tchesinkut Lake branch is shortened for better comparison of other branches (relative length of the whole network is shown inset). *Oncorhynchus nerka* images and population text color are as depicted in Fig 1. This figure was modified from [35].

We used previously published genotypic data at 7,347 SNPs [35] collected via restriction site-associated DNA sequencing (RADseq) from stream-, shore- and deep-spawning kokanee and anadromous sockeye salmon distributed across three geographic basins (Columbia, Skeena, and Fraser) in British Columbia. These systems encompass those where reproductive ecotypes co-occur (e.g., Okanagan Lake, Wood Lake), migratory forms overlap (e.g., Skaha Lake kokanee-Okanagan River sockeye; Anderson/Seton Lake kokanee-Portage Creek sockeye), and/or within-lake geographic population structure has been previously inferred (e.g., North versus West Arm Kootenay Lake kokanee) (Fig 1). This initial pool of candidate SNPs (hereafter referred to as *RADseq_kokanee*) included outlier loci that provide signal to differentiate migratory and reproductive ecotypes, as well as neutral loci that were previously filtered for putative paralogs and loci that deviated from Hardy-Weinberg equilibrium [35]. Divergence and relative relationships among the reference populations inferred from the original RADseq data are detailed in the phylogenetic network reproduced from [35] (Fig 2).

The initial step in panel design was to remove loci with insufficient flanking sequence for primer design, ensuring that the SNP of interest was located between the 40^th^ and 70^th^ base pairs of the contigs. Moving forward, this step will not be necessary, as the *O*. *nerka* reference genome can be used for this purpose, which was not yet available at the time of this study [37]. We calculated Weir and Cockerham’s (1984) unbiased estimates of pairwise genetic differentiation (*θ*) as implemented in VCFtools [38, 39] across different groupings in *RADseq_kokanee*. We then selected a total of 550 informative SNPs from a sequentially ranked list by *θ* values for each comparison based on previous trends of genetic divergence, with some loci exhibiting high *θ* across multiple comparisons: 1) geographic basin (Columbia v. Fraser v. Skeena) (200 SNPs); 2) migratory form (anadromous sockeye v. resident kokanee) (100 SNPs); and 3) kokanee reproductive ecotype (stream- v. shore- v. deep-spawning) (250 SNPs) (Table 1 and Fig 1). All loci pairs were assessed for deviation from linkage equilibrium using a Fisher’s Exact Test as implemented in GENEPOP version 4.7 [40]; in cases of significant association, we removed the less informative locus after correction for false discovery rate using the Benjamini-Hochberg procedure [41].

**Table 1:** Kokanee and anadromous sockeye salmon samples used for assignment to migratory form and reproductive ecotypes across historical drainages.

Location	Migratory Ecotype	Reproductive Ecotype	Historical Drainage	Reference Sample Size	Novel Sample Size
Wood Lake	Kokanee	Shore	Columbia River	34	4
	Kokanee	Stream	Columbia River	34	4
Kootenay Lake	Kokanee	Shore (West Arm)^a^	Columbia River	45	5
	Kokanee	Stream (West Arm) ^a^	Columbia River	48	3
	Kokanee	Stream (North Arm)	Columbia River	22	3
Okanagan Lake	Kokanee	Shore	Columbia River	48	12
	Kokanee	Stream	Columbia River	48	8
Skaha Lake	Kokanee	Stream	Columbia River	20	9
Okanagan River	Sockeye	Stream	Columbia River	35	10
Portage Creek	Sockeye	Stream	Fraser River	23	3
Anderson Lake	Kokanee	Deep^b^	Fraser River	23	4
Seton Lake	Kokanee	Deep^b^	Fraser River	23	4
Tchesinkut Lake	Kokanee	Shore^c^	Skeena River	36	4
	Kokanee	Stream^c^	Skeena River	36	4

The following reference populations were pooled for assignment analysis due to lack of structure in these systems as found in [35]:

^a^ Kootenay Lake West Arm shore- and stream-spawning kokanee;

^b^ Anderson Lake and Seton Lake deep-spawning kokanee;

^c^ Tchesinkut Lake shore- and stream- spawning kokanee. ‘Reference Sample Size’ represents the number of individuals included in baseline reference populations from *RADseq_kokanee*. ‘Novel Sample Size’ represent novel individuals not included in the reference population.

To evaluate our initial pool of candidate loci, we used the Bayesian clustering method implemented in STRUCTURE 2.3.3 [42] to test their ability to reveal the same inferred number of genetic units as *RADseq_kokanee* from [35]. We used a run length of 200,000 MCMC iterations after a burn-in period of 100,000. The analysis was carried out using correlated allele frequencies under a straight admixture model and the LOCPRIOR option. The most likely number of clusters was determined using the Δ*K* [43] method as implemented in STRUCTURE HARVESTER [44]. The optimal value of *K* was then combined across ten independent runs with CLUMPP [45]. We also assessed individual assignment accuracy with the Rannala and Mountain (1997) estimator as implemented in ONCOR with 100 simulated individuals per population based on empirical genotype frequencies with *recom-sim* [46–48]. After assessment, we sent the full RAD tag sequences associated with the pool of candidate SNPs to GTseek LLC (https://gtseek.com/) for custom locus-specific primer design.

### GT-seq test library preparation

We constructed a GT-seq test library with previously extracted DNA samples from [35] that were not included in the *RADseq_kokanee* baseline in order to minimize high-grading bias. We estimated assignment accuracy (Table 1), including shore- (n = 25), stream- (n = 30) and deep-spawning (n = 8) kokanee across the Columbia, Fraser and Skeena River basins, as well as anadromous sockeye salmon from the Columbia (n = 10) and Fraser River basins (n = 3). Additionally, we included repeated samples from the *RADseq_kokanee* baseline (n = 10) to assess genotyping error between RADseq and GT-seq, with five baseline samples repeated to assess within GT-seq genotyping error (Table 1).

Extracted DNA was quantified with a Qubit 3.0 Fluorometer and the Qubit dsDNA High Sensitivity DNA Quant Kit (Invitrogen). Library preparation followed the standard GT-seq protocol [18], with the exception that we diluted the PCR1 product to 1:10 (doi.org/10.17504/protocols.io.byvppw5n). The PCR2 product was quantified with Picogreen^TM^ (Molecular Probes, Inc.) and each sample was normalized to a concentration of 10 ng/μL. The pooled library was purified with a MinElute PCR Purification Kit (Qiagen) and eluted into a final volume of 25μl. Test libraries were sequenced using a single lane of Illumina MiSeq paired-end 150 bp sequencing at the McGill University and Genome Quebec Innovation Centre.

### GT-seq genotyping and primer optimization

Demultiplexed raw sequencing files were processed with the GT-seq pipeline available on GitHub (https://github.com/GTseq/GTseq-Pipeline). We removed loci that met these criteria: 1) primers with unequal counts indicating non-specificity of *in silico* probes; 2) candidate loci exhibiting >2% of the raw read count; 3) loci with no counts indicating off-target amplification or *in silico* probe variation; 4) loci contributing to potential PCR artefacts; and 5) observed primer dimers. A second test library was prepared with an optimized primer pool and the same samples as library 1 according to the protocols detailed above for sample preparation, sequencing, and primer dropout.

Raw sequencing files for the same individual were concatenated across libraries. Genotypes were called and compiled into one file that was converted to a .ped file with *GTseq_ped_converter3*.*py* (https://github.com/schmanii/GT-seq). Individuals and loci with >30% missing data were removed from downstream analyses with PLINK [49]. We then calculated genotyping error rates within and among RADseq and GT-seq sequences by comparing individual genotypes between replicate samples with the custom script *genoerrorcalc*.*py* (https://github.com/bsjodin/genoerrorcalc).

### Self-assignment analysis of panel utility

We conducted self-assignment tests using the leave-one-out procedure and the Rannala and Mountain (1997) likelihood estimation method as implemented in ONCOR [46, 47]. Additionally, we employed a machine-learning framework using the Support Vector Machine algorithm with Monte-Carlo cross-validation using the R package assignPOP [50] to evaluate the reliability of genetic datasets for population assignment and to assess panel informativeness for assignment of individuals to geographic basin, migratory form, reproductive ecotype and geographic location at multiple scales. Analyses were run with 30 iterations at varying proportions of the top *θ* loci (50%, 75%, 100%) and varying proportions of training individuals (50%, 90%).

### Assignment of novel individuals across British Columbia

We filtered genotypic data by individual and locus with a 30% missing data threshold in PLINK [49] to accurately identify individual populations that may have stronger signals from other comparisons (e.g. basin signal stronger than an ecotype signal). Individuals were then assigned to *RADseq_kokanee* pure stock populations with the method of Rannala and Mountain (1997) as implemented in ONCOR and the support vector machine algorithm as implemented in assignPOP [46, 47, 50].

### Wood Lake kokanee case study

To compare assignment results across methods, we selected previously extracted DNA samples from [7] that were previously genotyped at 10 microsatellite loci (2012 trawl survey, n = 105) and at 24 SNP loci via TaqMan^®^ genotyping assays (2014 angler survey, n = 84). We genotyped these samples using the final optimized GT-seq panel and a Mid Output Reagent Kit (300 cycles) on an Illumina MiniSeq.

To extend an annual time-series conducted for Wood Lake since 2012, operculum punches were collected from angler surveys during the spring in 2018 (n = 65) and 2019 (n = 100). These samples were collected by personnel from the British Columbia Ministry of Forests, Lands, Natural Resource Operations and Rural Development, which is the management authority charged with issuing permits and managing freshwater fisheries in the Province. Collection procedures also adhered to University of British Columbia Animal Care protocol # A19-0103. We extracted DNA with Chelex (doi.org/10.17504/protocols.io.byvhpw36) and constructed a GT-seq library with the final optimized panel and the methods detailed above.

The Wood Lake samples were sequenced using a partial lane of Illumina HiSeq paired-end 150 bp sequencing at the McGill University and Genome Quebec Innovation Centre. All individuals were genotyped by locus and individual, and filtered at a 50% missing data threshold in PLINK [49], as the identification of reproductive ecotypes within this system was sufficient at this threshold and allowed for the retention of samples that may have missing data due to lower quality DNA. In order to stay consistent with the stock assignment approaches used in the long-term time-series [7], we calculated the probability that an individual belongs to Wood Lake shore- or stream-spawning kokanee using the reference populations from the *RADseq_kokanee* baseline and the Rannala and Mountain (1997) method in ONCOR [46, 47]. In addition, we used a Bayesian method to assign individuals to Wood Lake shore or stream-spawning kokanee as implemented in STRUCTURE 2.3.3 [42, 43]. We used a run length of 1,000,000 MCMC replicates after a burn-in period of 500,000. The analysis was carried out using correlated allele frequencies under a straight admixture model and the LOCPRIOR option for the use of sampling location information to delineate closely related populations. The maximum number of clusters was set at *K* = 2 to assign individuals to the stream or shore cluster following [7]. Membership coefficients for each sample were averaged across 10 replicates with CLUMPP 1.1.2 [45]. Individuals were assigned to the shore- or stream-spawning ecotype if membership coefficients were >0.80 following [51].

Mixed stock proportions were estimated for each year with 95% confidence intervals calculated from 10,000 bootstrap replicates using the conditional maximum likelihood-based approach as implemented in ONCOR and reference baseline data from pure stock Wood Lake shore- (n = 33) and stream-spawning (n = 34) kokanee in *RADseq_kokanee* [47].

## Results

### Panel optimization

The finalized SNP pool (n = 547) before primer design included those that exhibited the highest divergence across geographic basin (n = 102), migratory form (n = 87), reproductive ecotype (n = 182), or overlapped across multiple comparisons (n = 176). Bayesian clustering analyses conducted with the initial 547 candidate loci revealed an optimal value of *K* = 8, mirroring results conducted from the full set of neutral SNPs from [35]. Individual assignment accuracy of 100 simulated individuals per population was >99%.

**Fig 3:**
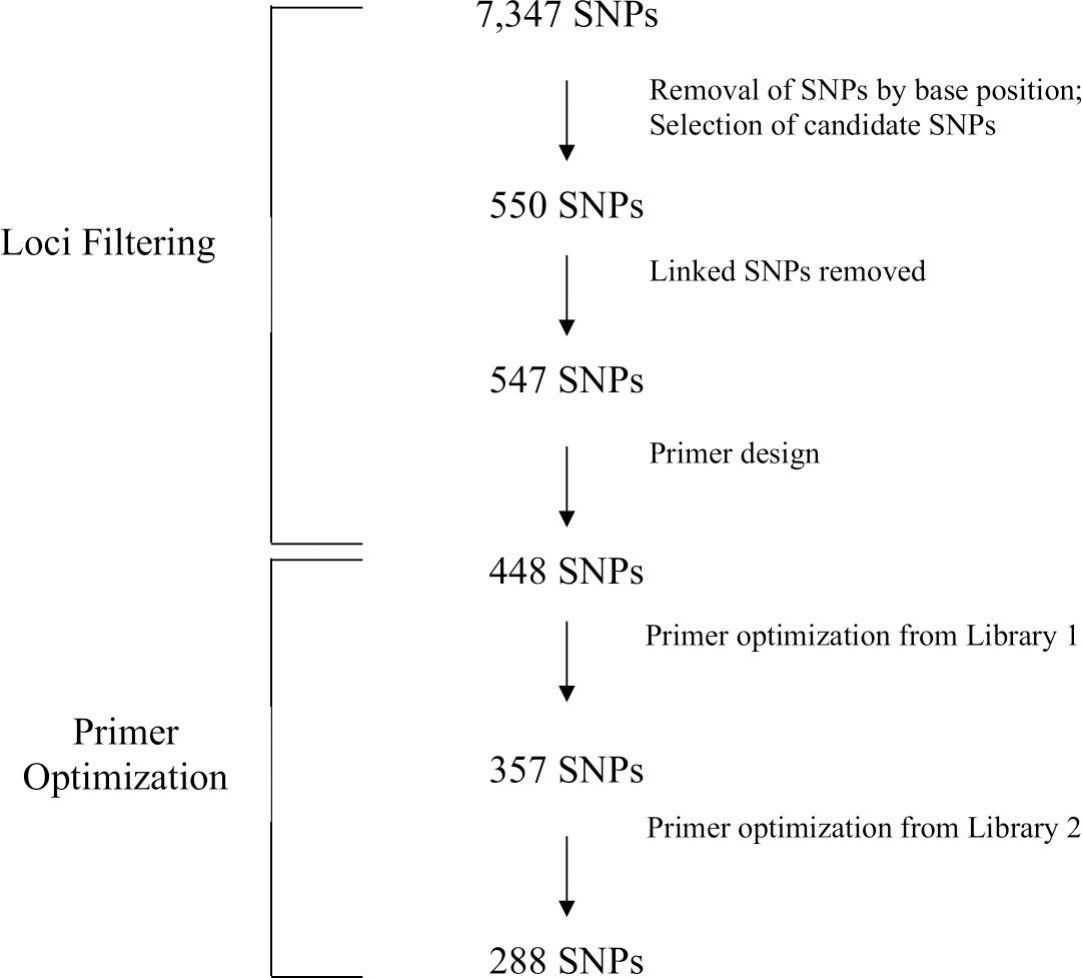
Workflow of initial *Oncorhynchus nerka* multi-use panel design and primer removal.

Of the 547 candidate SNPs, primers were successfully designed for 448 loci after *in silico* testing. Following multiplex amplicon sequencing and subsequent primer pool optimization, we retained a final optimized panel of 288 SNPs (S4 Table) consisting of loci informative at the following scales: geographic basin (n = 49), migratory form (n = 46), reproductive ecotype (n = 87), and across multiple scales [n = 106 total consisting of: geographic basin and migratory form (n = 17); geographic basin and reproductive ecotype (n = 51); migratory form and reproductive ecotype (n = 21); geographic basin, migratory form and reproductive ecotype (n = 17)] (Fig 3). Sixty of these loci were outliers previously detected for pairwise ecotype comparisons across the province [35]. We found the average genotyping discordance at 288 loci within GT-seq (n = 3) to be 1.07% and between RADseq and GT-seq (n = 5) to be 0.81%.

### Self-assignment and province-wide panel accuracy

**Fig 4:**
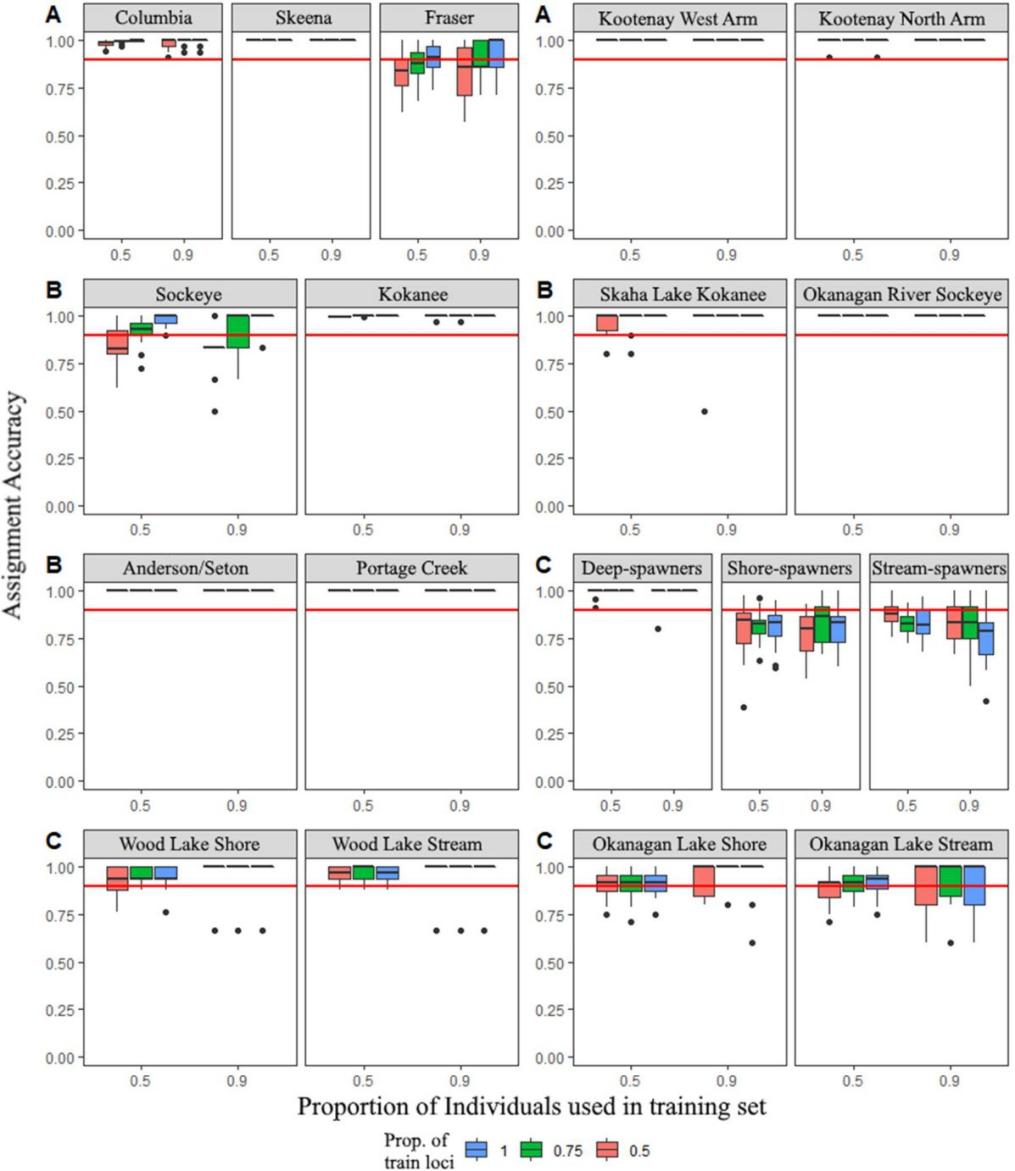
Assignment accuracies estimated using the support vector machine algorithm with Monte-Carlo cross-validation implemented in assignPOP [50]. Sampling of subsets of high *θ* loci (all loci, blue; top 75%, green; top 50%, orange) are crossed by two levels of training individuals (50%, 90%). Outliers are shown as black circles. The horizontal red line indicates 90% assignment rate. Results grouped by: A: geographic basin, B: migratory form, C: reproductive ecotype.

The optimized GT-seq panel exhibited a self-assignment accuracy of 97.7% based on leave-one-out tests using the Rannala and Mountain (1997) estimator [46]. The machine-learning model as implemented in assignPOP [50] revealed an average self-assignment accuracy of 93.3% when the analysis was conducted with every population analyzed simultaneously at all loci and 90% proportion of individuals. Self-assignment for comparisons by geographic basin, migratory form and system-specific comparisons mostly exhibited >90% assignment accuracy when using ≥0.75 individuals and ≥0.75 loci in the training set (Fig 4). The exceptions were comparisons by reproductive ecotype pooled as deep-spawning kokanee (Anderson and Seton Lakes), shore-spawning kokanee (Wood Lake, Okanagan Lake, Tchesinkut Lake) and stream-spawning kokanee (Wood Lake, Okanagan Lake, Skaha Lake, Kootenay Lake North Arm), which exhibited slightly lower assignment accuracies when using 0.70 proportion of individuals and 0.75 of the loci in the training set, but none were <0.80 (Fig 4).

### GT-seq British Columbia individual assignment

After filtering for missing data, we retained 56 novel individuals at 261 loci (average read depth = 117.8; genotyping rate = 90.1%). Assignment accuracy was 100% with the Rannala and Mountain (1997) estimator, and 98% with the support vector machine algorithm to the correct geographic location, migratory form, and reproductive ecotype (S1 Table).

### GT-seq SNP genotyping Wood Lake

We successfully genotyped Wood Lake individuals at 242 SNPs after filtering for missing data (average read depth = 177.5; genotyping rate = 93.3%) that were previously analyzed in [7] using microsatellites (2012 trawl; n = 99) and TaqMan^®^ SNP genotyping assays (2014 angler caught; n = 51). Overall assignment to Wood Lake shore- or stream-spawning kokanee was 99.3% concordant between ONCOR [47] and STRUCTURE [42] across all samples (S2 Table). Additionally, ecotype assignment based on the GT-seq panel was 98.0% concordant with previous designations at 24 SNPs and 98.9% concordant with samples previously assessed at 10 microsatellites. Four samples that were not confidently assigned originally using the 10 microsatellites (i.e. ecotype assignment was discordant between ONCOR and STRUCTURE) were assigned with high confidence to shore- (n = 1) and stream-spawner (n = 3) (S2 Table).

We successfully genotyped Wood Lake individuals from 2018 (n = 62) and 2019 (n = 92) at 282 SNPs after filtering for missing data (average read depth = 591.2; genotyping rate = 92.9%). Individual assignment was 98.7% concordant between ONCOR [47] and STRUCTURE [42] across all samples across years (S3 Table). Mixed-stock proportions varied between years, revealing shore-spawner proportions of 0.3893 (95% CI = 0.274–0.517) in 2018, and 0.2080 (95% CI = 0.131–0.304) in 2019, results that were highly consistent with the proportions of shore-spawners estimated using individual assignment in 2018 (0.390) and in 2019 (0.219) (S3 Table).

## Discussion

The ability to accurately differentiate life history variants such as migratory form and reproductive ecotype is vital for estimating fisheries management parameters such as recruitment and mortality in mixed-stock fisheries [10, 52–54]. To our knowledge, this is the first GT-seq panel demonstrated to simultaneously provide effective stock identification for a large number of populations spanning across broad-scale geographic basins to finer-scale life history variants such as migratory form and reproductive ecotypes, even in locations where they co-occur. The performance of this panel was demonstrated by way of high self-assignment accuracy (93.3%), high assignment accuracy (>98%) of novel individuals to their corresponding location, migratory form and reproductive ecotype, as well as contributing rapid, accurate and scalable stock identification to a long-term time series in Wood Lake, where stream- and shore-spawning kokanee co-occur. More broadly, our results highlight important considerations when applying this panel and developing those for other species and systems.

Regarding initial panel development and optimization, selection of an appropriate population assignment method is important for determining panel composition and overall utility. For example, assignment results from the leave-one-out approach (self-assignment: 97.7%; assignment of unknown individuals: 100%) with the Rannala and Mountain (1997) estimator [46] were notably higher than that of a machine-learning double-cross validation framework in assignPOP [50] across all populations using the same reference dataset (self-assignment 93.3%; assignment of novel individuals: 98%), highlighting the tendency for the former to suffer from a systematic upward bias in predicted accuracy [55]. This is an important consideration, as overestimated accuracy can subsequently lead to misidentification and inaccurate estimates of stock size, ultimately affecting long-term sustainability in mixed-stock fisheries, especially in closely-related populations [56]. Additionally, the training- and test-set protocol implemented in assignPOP [50] contains intrinsic sensitivity analyses to examine loci efficacy. Alternative approaches, such as those implemented in rubias [57], may also be worth exploring in this context, offering the potential to minimize assignment bias and provide complementary opportunities for cross-validation. Our methodology for candidate loci selection considered the level of genetic divergence before determining a targeted number of markers needed to effectively discriminate between populations of interest [58]. Candidate loci in the finalized 288 primer panel were initially selected based on their high *θ* signatures for reproductive ecotype, geographic basin, migratory form, or a combination of these categories. However, self-assignment accuracy from the machine learning framework was consistently >90% when analyzing data from the entire panel simultaneously, regardless of the specific level at which information was required; for example, inclusion of loci that were initially selected based on high *θ* at the among-geographic basin level did not reduce assignment accuracy between co-occurring reproductive ecotypes within single systems (e.g., Wood Lake). Although this cross-category signal may not necessarily be the case for multi-scaled SNP panels developed in other species or systems, the ability to simultaneously use the full set of 288 SNPs provides enhanced power due to an increased number of loci while limiting filtering steps and facilitating downstream analyses.

Though use of all loci in the panel can increase assignment accuracy, we found that population composition can affect panel resolution. For instance, grouping individuals from separate drainages in the same analysis can simplify the bioinformatic analysis of large-scale assessments, but runs the risk of leading to inaccurate results. Here, we found that the reproductive ecotype self-assessment accuracy at 90% training individuals and 100% training loci between shore-, stream-, and deep-spawning kokanee across British Columbia (86.7%) may have been affected by noise due to the grouping of ecotypes across geographic basin within this analysis. This result could indicate that the genetic signature of geographic basin across the province is stronger than that of reproductive ecotype, where shore- and stream-spawning kokanee populations within the same lake were found to be more closely related to each other than to their corresponding ecotypes in other lakes [35]. The reproductive ecotype accuracy markedly improved when Okanagan Lake (shore and stream = 94.0%), Wood Lake (shore and stream = 95.0%), and Anderson/Seton Lakes (deep-spawners = 100.0%) were analyzed separately. Depending upon the level of accuracy required for a specific application, GSI may be most effective using reduced subsets of individuals informed by an *a priori* assumption of location to provide system-specific signal to stock assignment, especially in mixed stock fisheries (e.g., Okanagan Lake kokanee; Fig 4C).

Size of initial reference population baselines is another important consideration in GSI that can be effectively addressed using data from GT-seq. Minimum baselines for GSI have been suggested with increased sample sizes found to improve resolution in closely-related groups [59]. GT-seq data combined with population assignment analyses implemented in assignPOP [50] can be used to inform minimum sample sizes needed for effective baselines in lake systems that span a variety of evolutionary histories. For example, low levels of genetic mixing between reproductive ecotypes in Okanagan Lake may reduce assignment accuracy. Here, we found that self-assignment of Okanagan Lake shore/stream-spawners improved with more test individuals (50% test individual level = 92% accuracy; 90% test individual level = 94% accuracy) (Fig 4C). In contrast, no improvement was demonstrated in the Kootenay North /West Arm between the 50% and 90% test individual levels (100% accuracy at all levels), indicating that populations that are geographically separated, even in the same system, may not require as many baseline individuals for accurate assessment (Fig 4A) [35]. GT-seq therefore provides a scalable approach for adding to reference population baselines that is otherwise inefficient by other techniques such as RADseq [60].

Comparison of the same SNPs can also be difficult in RADseq due to the combination of restriction enzyme digestion variation and bioinformatic assignment of RADtags, even with alignment to a reference genome. Though capture probes have been integrated into RADseq protocols to allow for sequence capture (e.g. Rapture) [61], these methods still require more expensive, labour-intensive library preparation when compared to the rapid turnaround that GT-seq offers at a lower price ($15/sample versus $6/sample) [62]. The consistent targeted capture of specific loci with the use of forward and reverse primers in GT-seq further allows for longitudinal datasets processed by different researchers to be connectable without the use of specialized laboratory equipment required for Rapture [61]. With population diversity found to be comparable between the same samples genotyped with both RADseq and GT-seq, this panel can be used to improve existing pure stock baselines for kokanee and provides an advantage when considering a shift towards GT-seq informed GSI in other systems [63].

More generally, GT-seq panel designs have revealed potential trade-offs to consider depending on panel intent and species characteristics. For example, a large primer pool designed for optimal panel performance needs to be balanced with the increased likelihood for primer interaction as well as the cost of subsequent primer drop out [54]. This observation has led to varied design methodologies in published GT-seq panels intended for individual assignment that are tailored to the needs of each study system. Four separate 300 loci GT-seq panels improved the assessment of closely related Chinook Salmon populations within a single river drainage in Alaska, but subsequently led to a higher overall cost associated with primers [64]. A smaller, dual use 172 loci panel designed for population assignment and pedigree reconstruction for *O*. *nerka* in Alaska found that a minimum of 43 markers was required to achieve >90% accuracy for population assignment across two creeks, revealing potential lower limits needed for high accuracy [53, 54]. Comparatively, our British Columbia-wide GT-seq panel of 288 SNP loci has demonstrated >90% self-assignment accuracy of fourteen different populations representing three geographic basins, two migratory forms, and three reproductive ecotypes across the province, even in systems with co-occurring ecotypes expressing little to no neutral genetic divergence.

In addition to considerations related to GT-seq panel composition, optimization and quality control, the accuracy of any GSI tool in real-world applications is of paramount importance. Our multi-scaled GT-seq panel showed high accuracy (100%) to assign novel individuals using the entire *RADseq_kokanee* baseline to specific stocks spanning geographic basin, migratory form, and reproductive ecotypes across British Columbia. Moreover, we demonstrated a real-world application to Wood Lake, a valuable mixed-stock fishery of co-occurring shore- and stream-spawning kokanee that has been subject to long-term, genetic stock assessment using a range of different molecular markers, from expressed sequence tag-linked microsatellites to TaqMan^®^ SNP genotyping assays [7]. Shore-spawning kokanee are especially difficult to discern from co-occurring stream-spawners using traditional visual count methodologies due to their general lack of morphological distinction [28, 35], as well as the in-lake low visibility conditions and wide-ranging habitat area in Wood Lake specifically [7]. Recent visual surveys have reported <100 shore-spawners in the system, with declines precipitated by anoxic conditions and lethal water temperatures [7]. Previous findings conducted with 24 microsatellites revealed that the shore-spawner population in Wood Lake was four times greater than previously estimated by visual counts from 2014–2016 [7]. With our GT-seq panel, we found the proportion of shore-spawners in 2018 (0.39) and 2019 (0.21) to be consistent with previous trends in the system [7]. Moreover, we were able to assign four samples that were ambiguous with conflicting assignment between ONCOR and STRUCTURE based on earlier microsatellite-based GSI to a specific ecotype, reinforcing the ability of dense SNP panels to provide finer-scale resolution compared to more traditional markers [12]. Additionally, the high GSI concordance (>98%) between different classes of molecular markers (e.g., microsatellites and SNPs) and additional power provided by the GT-seq panel can be leveraged to bolster previous assessments if warranted in the future.

Overall, our results demonstrate that this multi-scaled GT-seq panel is highly informative for guiding kokanee fisheries management within the systems included in the baseline database (*RADseq_kokanee*). Although it remains unclear the degree to which this panel can be applied to systems outside of British Columbia, at minimum we expect these markers to have the capacity to identify individuals to migratory form and reproductive ecotype given the inclusion of SNPs found to be informative for this purpose from previous studies [34, 35]. More broadly, the design methodology and multiple levels of robust quality control demonstrated here can be applied for informing the development of panels for other systems and species with multiple levels of genetic variation that would benefit from GSI. GT-seq panels are emerging as valuable GSI tools for informing a range of management applications such as ongoing monitoring of critically depleted fish stocks [65], inferences on migration [66], conservation unit delimitation [67], and invasive species management [68]. Here, we have demonstrated the effectiveness of the British Columbia *O*. *nerka* GT-seq panel to inform temporal and spatial monitoring of mixed-stock kokanee fisheries moving forward.

## Supporting information

S1 Table GT-seq British Columbia individual assignment. (XLSX)

S2 Table GT-seq Wood Lake assignment accuracy. Previous assignments indicated in greyed boxes. Red text indicates ambiguous assignment, blue text indicates confirmed assignment for previously ambiguous assessment.
(XLSX)

S3 Table GT-seq 2018 and 2019 Wood Lake assignments. Red text indicates ambiguous assignment.
(XLSX)

S4 Table GT-seq primer sequences for the optimized British Columbia *O*. *nerka* GT-seq panel based upon Campbell et al. (2015) and IDT for Illumina TruSeq UD adapter sequences. (XLSX)
